# A Case Study of Suspected Childhood Apraxia of Sign

**DOI:** 10.1044/2024_persp-24-00042

**Published:** 2024-08-07

**Authors:** Christyn Jackson, Lauren Hagstrom, Karen Emmorey

**Affiliations:** a School of Speech, Language, and Hearing Sciences, San Diego State University, CA; b Independent Researcher and Speech-Language Pathologist, San Diego, CA

## Abstract

**Purpose:**

We provide a case report of “Zoe,” a 4-year-old deaf child from a deaf signing family, who presented with a possible case of Childhood Apraxia of Sign (CASign).

**Method:**

The description is based on reports from the child’s speech-language pathologist, her Individualized Education Program report, and a clinician-created sign imitation task.

**Results:**

Zoe’s sign articulation errors in American Sign Language differed from those reported for typically developing deaf children and were parallel to the types of errors observed for hearing children with childhood apraxia of speech. Specifically, Zoe produced inconsistent errors across signs, substituted more complex handshapes for simple handshapes, made errors on both unmarked (common) and marked (less common) forms, produced articulatory distortions (rather than substitutions), and exhibited “groping” behaviors (a sequence of attempts to move her hands into the correct position). In addition, Zoe sometimes self-corrected her errors by manipulating her own hands, for example, using her left hand to move the thumb of her right hand into the correct position.

**Conclusion:**

Zoe’s pattern of sign errors is consistent with an underlying deficit in motor planning and/or programming and may constitute the first reported case of CASign.

The field of speech-language pathology has focused on spoken language disorders and emphasized monolingual, English-speaking, hearing children (e.g., Guiberson & Atkins, 2012). There is limited research on language and articulation disorders in deaf, signing children, which poses a critical problem for professionals who want to implement evidence-based approaches to support sign language development. There have been a few studies of developmental language disorder (DLD), which described characteristics of DLD in deaf, signing children, and the observed linguistic patterns were both parallel and distinct from DLD in hearing, speaking children (e.g., Mason et al., 2010; Quinto-Pozos et al., 2017). However, to our knowledge, there have been no studies of possible childhood apraxia in sign language, which would be parallel to childhood apraxia of speech (CAS). Here, we report the case of a 4-year-old deaf child whose first language is American Sign Language (ASL) and who presented articulation difficulties that parallel the oral articulatory symptoms of CAS, which we have characterized here as Childhood Apraxia of Sign (CASign).

For spoken language, CAS is typically defined as a disorder impacting motor planning and programming of speech (American Speech-Language-Hearing Association [ASHA], 2007). Speech intelligibility is reduced due to an impairment in the transformation of abstract phonological representations into motor speech commands (Terband et al., 2009). CAS is diagnosed using a variety of speech tasks to differentiate a deficit in motor planning from a phonological impairment. The hallmark features of CAS generally include inconsistent errors on consonants and vowels across repeated productions, lengthened or disrupted co-articulatory transitions between sounds, groping movements of the articulators, and prosodic abnormalities (ASHA, 2007). CAS frequently co-occurs with other speech and language disorders (Iuzzini-Seigel et al., 2022) and commonly occurs in children with autism (Allison et al., 2020). CAS is also known to co-occur along with other motor disorders such as oral apraxia, limb apraxia, and developmental coordination disorder (Iuzzini-Seigel et al., 2022).

Acquired apraxia (specifically, ideomotor limb apraxia) has been documented in deaf adults, poststroke (Helm-Estabrooks, 1984; Poizner et al., 1984). Apraxia is distinct from sign language aphasia and is characterized by deficits in copying and producing noncommunicative sequences of meaningless movements (e.g., moving an outstretched arm across the body while opening the fingers). Sign apraxia can be distinguished from a sign-based phonological deficit. Just as words are composed of phonological units (consonants and vowels), signs are composed of manual phonological units: handshape, place of articulation (location), and movement (see Brentari, 2019, for a review of sign language phonology). A phonological deficit can be characterized by mis-selections of these units (e.g., substituting one handshape for another; Corina, 2000), whereas sign apraxia is characterized by misarticulations and planning errors.

Because all children make errors as they acquire language, it is critical to distinguish typical production errors from errors that indicate a disorder. Children acquiring a sign language exhibit a common pattern of production errors that parallel some patterns found in speech. For example, more complex articulations (e.g., more complex handshapes or movements) are mastered later and are more error prone (Boyes Braem, 1990; Mann et al., 2010). Phonological complexity in a sign language has been defined (in part) by articulatory effort (Morgan et al., 2019; Sehyr et al., 2021). For example, handshapes that involve groupings of fingers, rather than all fingers (e.g., a 5 handshape) or just the index finger, are more complex, and hand configurations that involve “stacked” or “crossed” features (i.e., the K and R handshapes) are more complex. All handshapes mentioned in the text are illustrated in the Appendix. In typical development, more complex handshapes are substituted with less complex handshapes, and complex movements are simplified (Marentette & Mayberry, 2000; Morgan et al., 2007). For example, when producing a sign with both path movement (the hand moves from one location to another) and internal movement of the hand (e.g., finger wiggling), children often omit one of these movements. In addition, unmarked (simple, frequent) handshapes and movements that require the least amount of motor control are produced early in childhood and are found in manual babbling (Petitto & Marentette, 1991). Location is the easiest parameter for children to acquire and is mastered at the youngest age, with very few location errors after the age of 3 years (Conlin et al., 2000; Mann et al., 2010). As with spoken language, phonologically simple signs are produced earlier and more accurately than phonologically complex signs (Meier et al., 2008). In general, phonologically simple signs are one-handed with unmarked handshapes and a single movement type, while more complex signs are two-handed, have marked handshapes, and have both internal and path movements (see Sehyr et al., 2021, for examples and a metric for assessing the phonological complexity for signs).

What might CAS look like in the manual modality? We assume that the parallel deficit would involve an impairment in the transformation of abstract phonological representations of signs into motor commands for the hands and arms. The phonological representations of signs should generally be intact, that is, the child has acquired an accurate mental representation of the form of signs and knows the target form of a given sign. Therefore, we should not observe systematic substitutions of phonological units such as handshape or movement substitutions across signs (e.g., regularly substituting a tapping movement for a twisting movement) or a consistent misarticulation of a target sign (e.g., consistently producing the ASL sign DOLL^1^ at the lips, rather than at the nose). Rather, the errors we observe with CASign should reflect difficulty with motor programming and planning as evidenced by inconsistent errors across sign productions, although we note that some children with phonological disorders may produce inconsistent errors and some children with severe CAS may produce consistent articulation errors (Murray et al., 2015). We should also find evidence of manual “groping” and possibly unusual articulatory errors that are not characteristic of typical development (e.g., substituting a more complex handshape for a simple handshape or producing a handshape that does not exist in ASL). Much of the phonological structure of signs is simultaneous, rather than sequential; for example, most signs are monosyllabic (defined [in part] as one sequential path movement), and there are very few multisyllabic signs (Brentari, 2019). Therefore, examining the effect of sign length on articulation is not as useful in diagnosing CASign as it is for identifying CAS. In addition, there is no clear parallel to “voicing” in sign language phonology, so this type of error cannot be documented. On the other hand, there may be some behaviors that are specific to the manual modality that may be helpful in diagnosing CASign, such as the ability to easily manipulate one’s own articulators.

In the case study reported here, we describe the language characteristics and the pattern of errors produced by a deaf, signing child, which suggest a deficit in motor planning and a diagnosis of CASign. Institutional review board approval is not required for case studies based on existing reports. Zoe’s caregiver gave written consent for her reports to be studied and described in a publication.

## Case Description

This case description is based primarily on reports contained within the Individualized Education Program (IEP) for Zoe (a pseudonym), which included reports by her speech-language pathologist (SLP), deaf and hard-of-hearing teacher, occupational therapist, audiologist, and school psychologist. Zoe is a 4-year-old, deaf signing child who has had consistent exposure to ASL at home from her two deaf parents and at school in her signing, preschool classroom. All of the students in the classroom used ASL along with some spoken English, and the teacher was a native ASL signer. Zoe has a profound mixed hearing loss in her right ear and a mild-to-moderate sensorineural hearing loss in her left ear. She wore hearing aids in both ears regularly. Zoe is female, right-handed, and had no reported birth complications.

When observed in her classroom, Zoe exhibited developmentally appropriate social abilities and pragmatic language skills. She was cooperative in her preschool classroom and enjoyed playing with peers and adults. She demonstrated age-appropriate imaginative play skills and sequential play abilities (e.g., doing laundry). Zoe knew how to get a person’s attention by waving or vocalizing. She was also skilled at using gestures to communicate her intended message. For example, she initiated a game of hide-and-seek with a peer by tapping her friend’s shoulder, closing her eyes, and beginning to count on her fingers and pointing to communicate to her friend to go hide. Zoe regularly used joint attention, eye gaze, gestures, and pointing to communicate, although she struggled to produce both signs and spoken words at school and at home.

Zoe presented with an expressive/receptive language delay in both ASL and English, which can also accompany CAS in hearing children (ASHA, 2007; Lewis et al., 2004). Zoe’s spoken language consisted of only a few single word productions and no two-word combinations. Her speech was often imprecise and inconsistent. She was evaluated for CAS using dynamic assessment procedures (e.g., Glaspey & Stoel-Gammon, 2007; Strand & McCauley, 2019) by her ASL–English bilingual SLP and an SLP with additional training and expertise in CAS. During informal word repetition tasks, Zoe was asked to repeat CVCV words (e.g., mama, pony) with multiple attempts encouraged and cues provided by the SLP. Her spoken word repetitions contained vowel distortions, atypical substitutions (e.g., /m/ for /b/), and groping behaviors (e.g., biting her lip before attempting to imitate /m/). Zoe also exhibited an increase in the number of errors as the words increased in length and had difficulty sequencing sounds together. For example, a sound or syllable she could say in isolation was produced incorrectly when placed in a two-syllable word.

Zoe’s expressive ASL was stronger than her spoken English. She had a small repertoire of signs that she could regularly articulate correctly (e.g., EAT, BATHROOM, WANT), and her use of two sign combinations was emerging. Zoe also produced code-blends, or signs produced simultaneously with spoken words, which are typical for deaf and hearing bimodal bilingual children and adults (Emmorey et al., 2008; Lillo-Martin et al., 2014). Zoe did not produce much spontaneous signing and needed to be prompted to produce signs. Like some hearing children who exhibit CAS (e.g., Chenausky, Verdes, & Shield, 2022), Zoe produced imprecise signing that was difficult to understand, even for familiar communication partners such as her parents and deaf classroom teacher. Zoe misarticulated signs and made errors across the parameters of handshape, movement, and location (described in more detail below).

Zoe also presented with delays in receptive language abilities in both ASL and spoken English, although her receptive language skills were stronger than her expressive language skills, as may often occur for children with CAS (ASHA, 2007; Lewis et al., 2004; Rosenbek & Wertz, 1972). For sign language, Zoe followed simple classroom directions in ASL and answered simple questions (e.g., HEARING-AID WHERE?; “Where are your hearing aids?”). She followed ASL directions that contained two critical elements (e.g., BABY SLEEP; “put the baby to bed”), but she had difficulty with more complicated instructions that had more than two critical elements (e.g., “make mom eat the apple”). The ASL Receptive Skills Test (Allen & Enns, 2013), the only standardized test of ASL comprehension for children, aged 3–13 years, was attempted with Zoe. However, she had difficulty attending to the entire videos, which are the test stimuli. She also demonstrated that she did not understand most of the vocabulary used in the test videos. For spoken language, her SLP reported that Zoe understood common English words (e.g., “dad,” “mom,” “wash,” “yes,” “hi”), but her ability to understand simple spoken English sentences without ASL support was very limited. Standardized tests of English were considered inappropriate for Zoe, and her spoken language comprehension ability was based on observation and performance-based assessment.

With respect to nonlinguistic motor abilities, Zoe displayed appropriate gross motor skill (e.g., getting up from a chair, walking on varied surfaces, and bending to pick up objects). However, she had difficulty with fine motor activities (e.g., handwriting, stringing beads). She required assistance for some classroom activities such as cutting with scissors, and she used two hands for many tasks that are typically performed with one hand (e.g., picking up small objects).

Her linguistic motor abilities were assessed using an ASL articulation probe that was previously created by an ASL–English bilingual SLP with expertise in ASL assessment. For this articulation task, another clinician who also specialized in speech and sign language intervention modeled the ASL signs for Zoe to imitate. Dynamic assessment was used for this probe task (e.g., manual cues were provided by the SLP such as modeling the correct handshape or manipulating Zoe’s fingers; see Mann et al., 2022, for discussion of the use of dynamic assessment with deaf, signing children). A total of 63 signs were elicited, which sampled a variety of ASL handshapes, movements, and locations. Below, we describe the errors that Zoe made on this imitation task and errors that were observed during interactions at her signing preschool, which were described in the reports in her IEP. We highlight errors that may be indicative of CASign: location (place of articulation) errors, atypical handshape errors and distortions, groping behavior, and self-manipulation of the hands.

## Location Errors

Location errors may indicate motor planning deficits for children acquiring ASL, because few location errors are made after the age of 3 years in either spontaneous or imitated productions (Conlin et al., 2000; Mann et al., 2010). Location errors in children are argued to be rare, because the gross motor ability to reach toward a location is solidly in place by 1 year, and target locations constitute relatively broad categories that do not require fine distinctions for accurate production (Siedlecki & Bonvillian, 1993). However, Zoe demonstrated location errors more frequently than would be expected for a typically developing 4-year-old based on previous studies of signing children (Conlin et al., 2000; Mann et al., 2010; Siedlecki & Bonvillian, 1993). For example, when asked to repeat the sign ROSE, which is produced at the nose, Zoe produced the sign at her eyes. Although we interpret this error as evidence of a motor planning impairment, that is, difficulty in targeting the correct location on the face, it is nonetheless possible that this error could reflect an incorrect phonological representation of the location of the sign as at the eyes, rather than at the nose.

**Figure 1.**
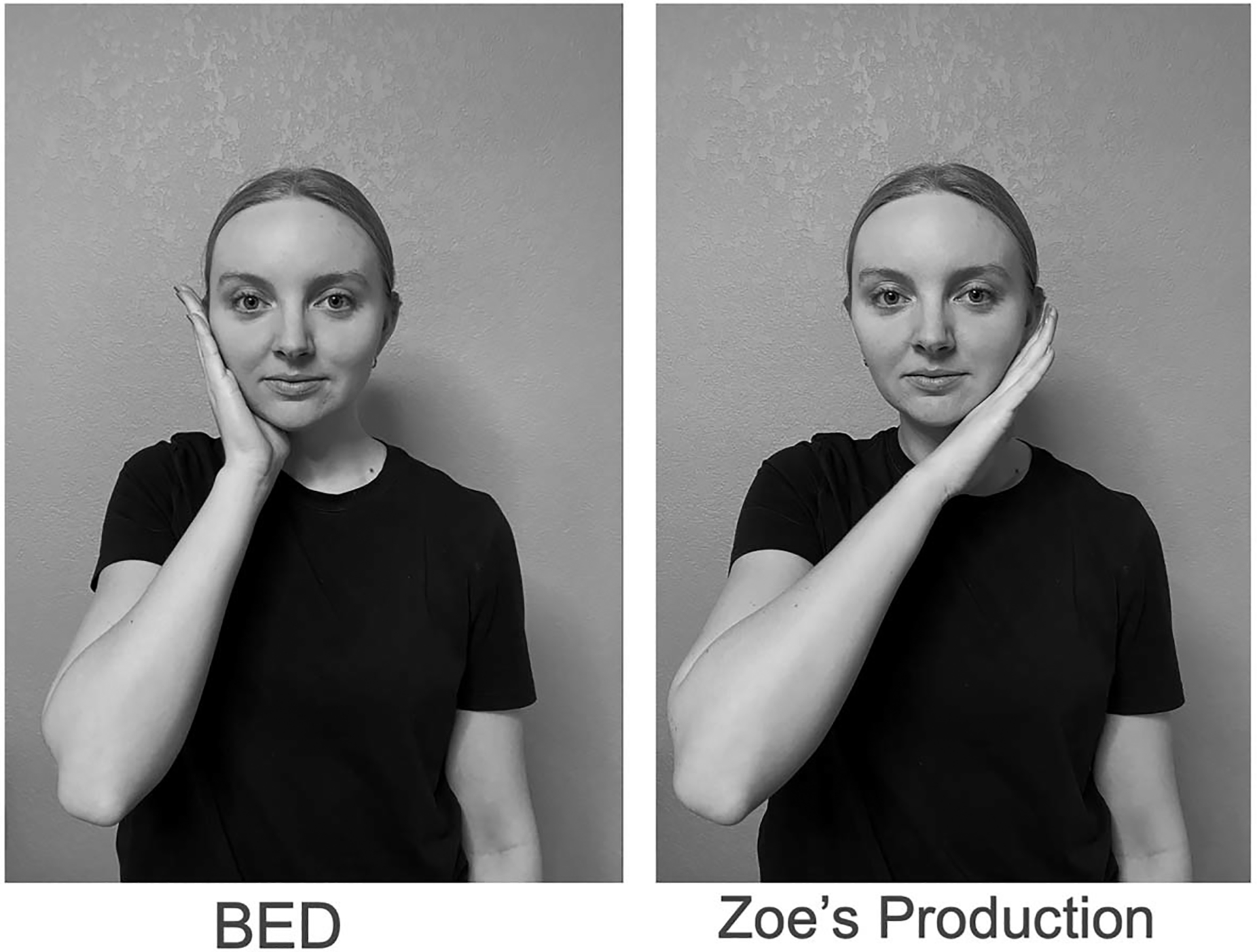
Example of a location error produced by Zoe that violates phonotactic constraints in American Sign Language.

In addition, Zoe produced location errors that violated phonotactic constraints in ASL. For example, ASL does not allow one-handed signs that only contact the contralateral side of the face. When asked to produce the sign BED, which is produced at the right cheek (for a right-handed signer), Zoe produced the sign at her left cheek (see Figure 1). Although some hearing children with phonological disorders produce errors that result in sounds that are not in the target language (e.g., ingressive sounds for English; Hrastelj & Knight, 2017), it is not clear how Zoe’s phonotactic error could be explained by erroneous allophonic rules at the phoneme level (as proposed for ingressive errors in speech), particularly because this type of location error was not systematic, as would be expected under an allophonic account.

Zoe’s location errors are particularly surprising, because she was given a model to imitate. When typical signing children are asked to repeat signs, they almost never produce a location error (Mann et al., 2010). Furthermore, because she had a direct model rather than producing these spontaneously (e.g., being asked to produce the sign BED in response to where a baby sleeps), these errors do not indicate that she had an incorrect phonological representation. Thus, we suggest that location errors for older signing children, particularly within an imitation task, could be a marker of motor planning impairments associated with CASign, but we also note that location errors do not clearly rule out a phonological disorder.

## Inconsistent and Atypical Handshape Errors

**Figure 2.**
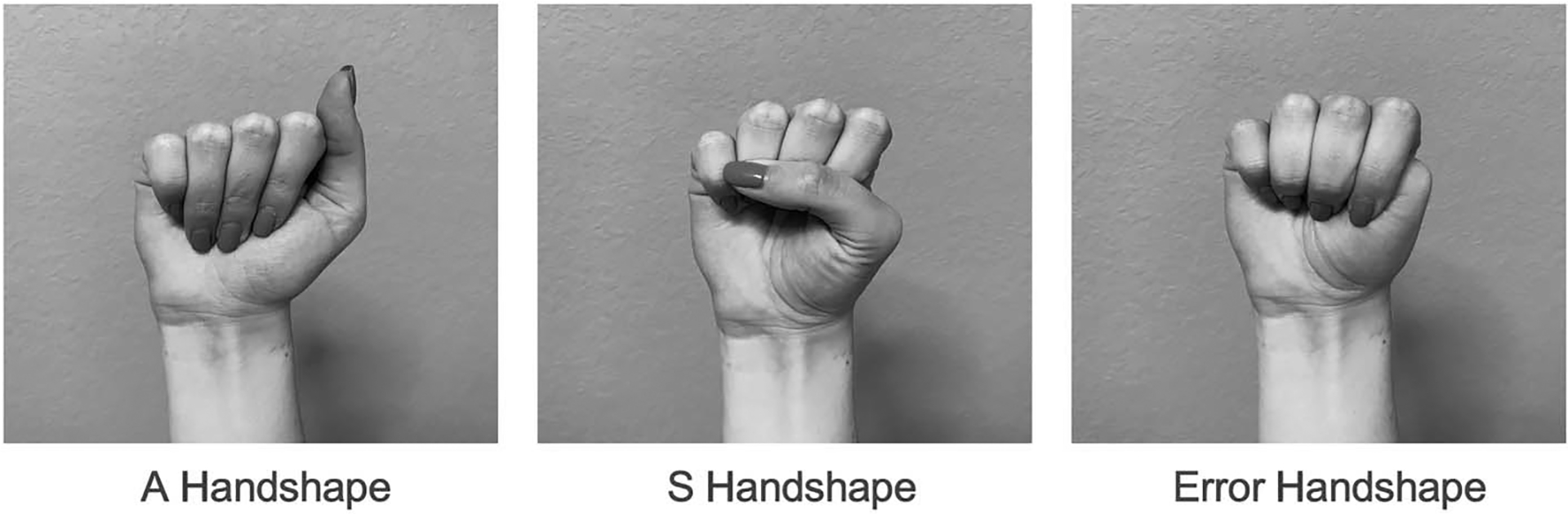
Illustration of the A and S handshapes that Zoe produced inconsistently when imitating the sign YES, and a handshape error produced by Zoe when copying the sign BICYCLE. The target handshape for both signs is the S handshape.

At age 4 years, Zoe should have mastered unmarked handshapes (i.e., commonly occurring, simple handshapes), but she continued to make errors on handshapes that are typically acquired very early, including the 1 handshape, the A handshape (a fist; see Figure 2), and the B handshape (all fingers extended and contacting each other). As with her location errors, her handshape errors were not systematic across signs. For example, for signs made with the S handshape (e.g., YES, BIKE), Zoe substituted three different (but similar) handshapes at three different times, and one of these handshapes was not a possible handshape in ASL (see Figure 2). At other times, Zoe was able to correctly produce unmarked handshapes; for example, she produced the target 1 handshape for the sign BLACK and the target A handshape for the sign WITH. Such inconsistent production errors are not indicative of a phonological disorder or of articulation difficulty with a particular phonological target.

**Figure 3.**
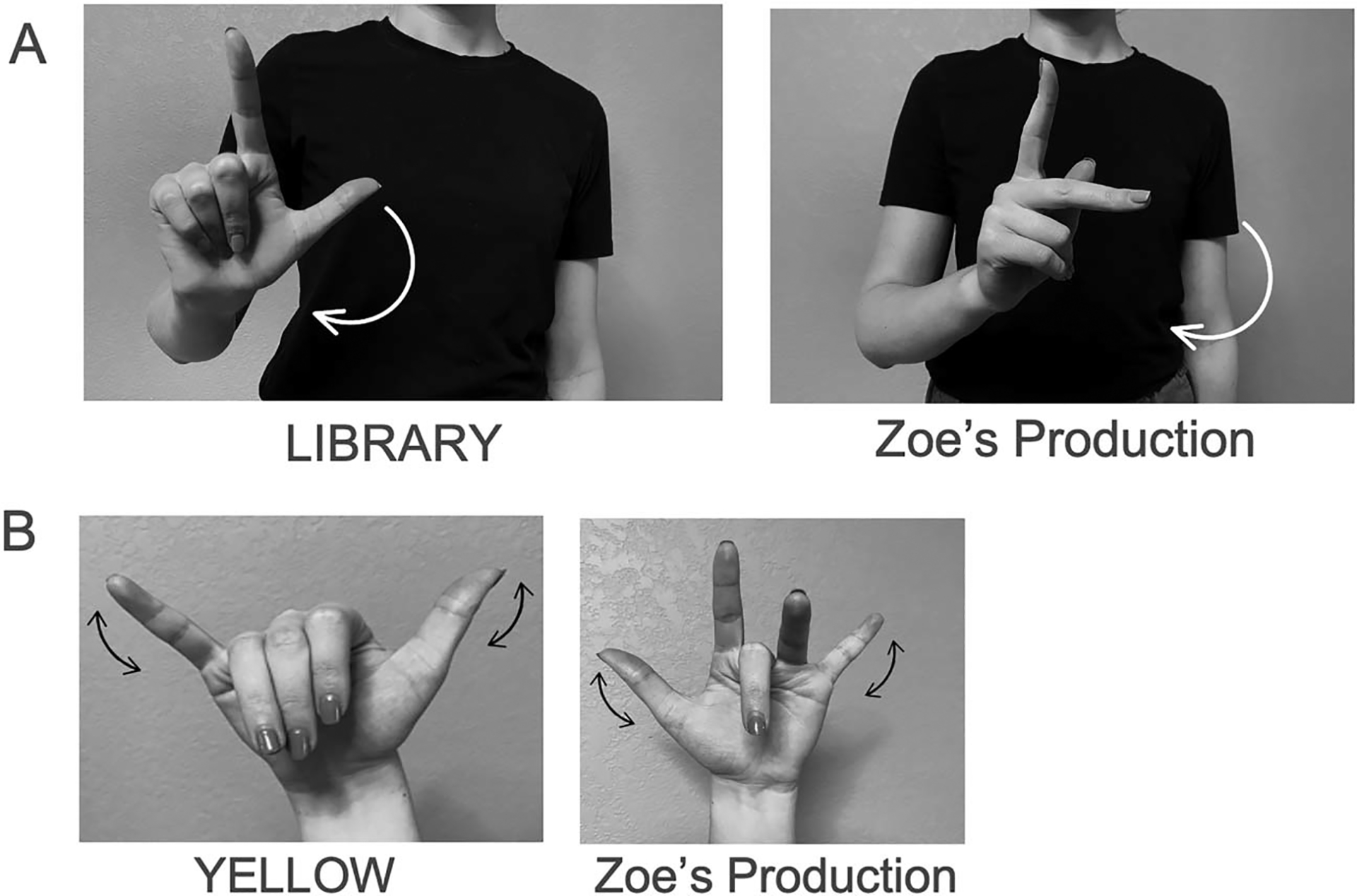
Example of Zoe’s substitution of more complex handshapes for simple handshapes in the signs LIBRARY (A) and YELLOW (B).

**Figure 4.**
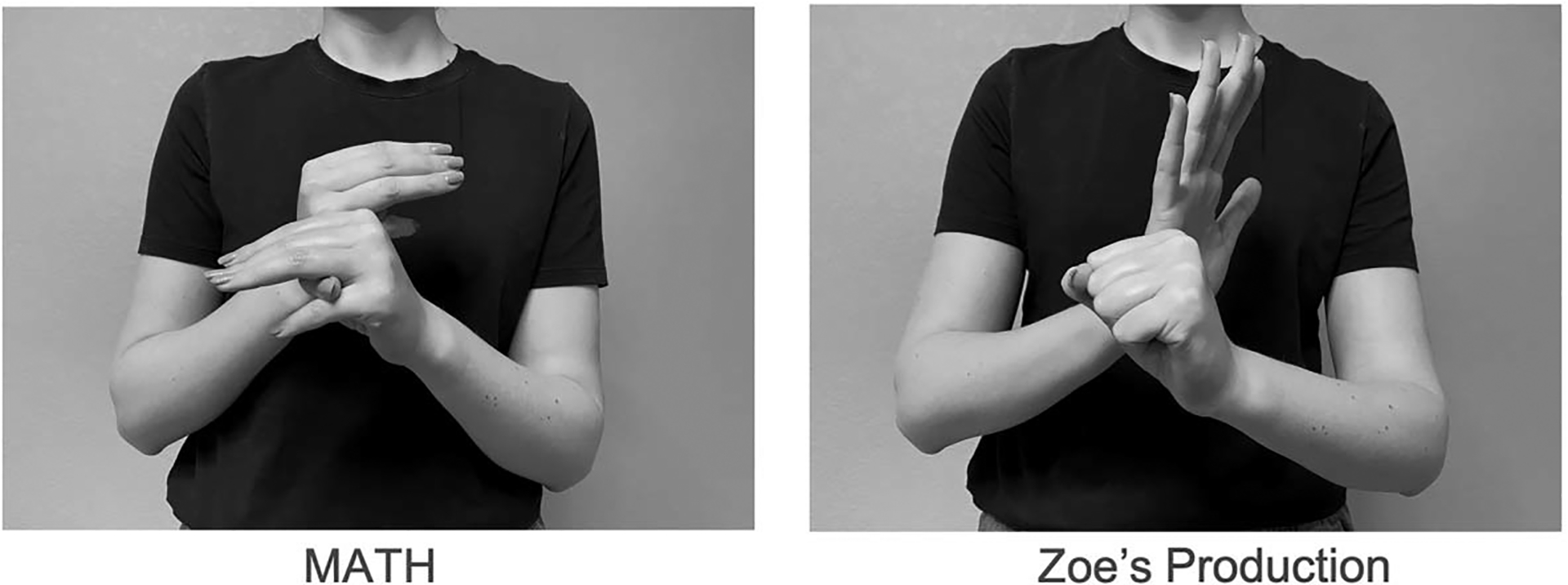
Example of an error that violates the phonotactic constraints on two-handed signs and that creates a more complex production.

Unlike typically developing signing children but paralleling hearing children with CAS, Zoe sometimes substituted more complex phonological forms for simple forms. For example, when imitating the sign LIBRARY, which is produced with a relatively simple L handshape, Zoe produced the sign with a K handshape, which is phonologically more complex (see Figure 3A). When asked to imitate the signs YELLOW and SILLY, she produced the complex open-8 handshape for the simpler target Y handshape (see Figure 3B). For two-handed signs, Zoe sometimes produced variations that are not allowed in ASL and that were also more complex (more difficult to produce). For example, when asked to imitate the sign MATH (produced with two M handshapes), Zoe produced the sign with an S handshape for one hand and a 5 handshape for the other hand (see Figure 4). Two-handed ASL signs with symmetrical movement (like MATH) must have the same handshapes on each hand (Battison, 1978), and thus this error violates a general phonological constraint on the form of signs. These handshape errors are very different from those produced by typical signing children and thus are not explained by a language delay (Gu et al., 2022). In addition, these errors are not easily explained by referring to phonological features, such as an error in selecting position features (open, closed) or flexion features (bent, flat) of the Selected Fingers of the handshape (those fingers that move or contact the body; Brentari, 2019). We suggest that these inconsistent and atypical handshape errors are best explained by an impairment in motor planning and organization of finger configurations during sign production.

## Handshape Distortions and Groping Behavior

**Figure 5.**
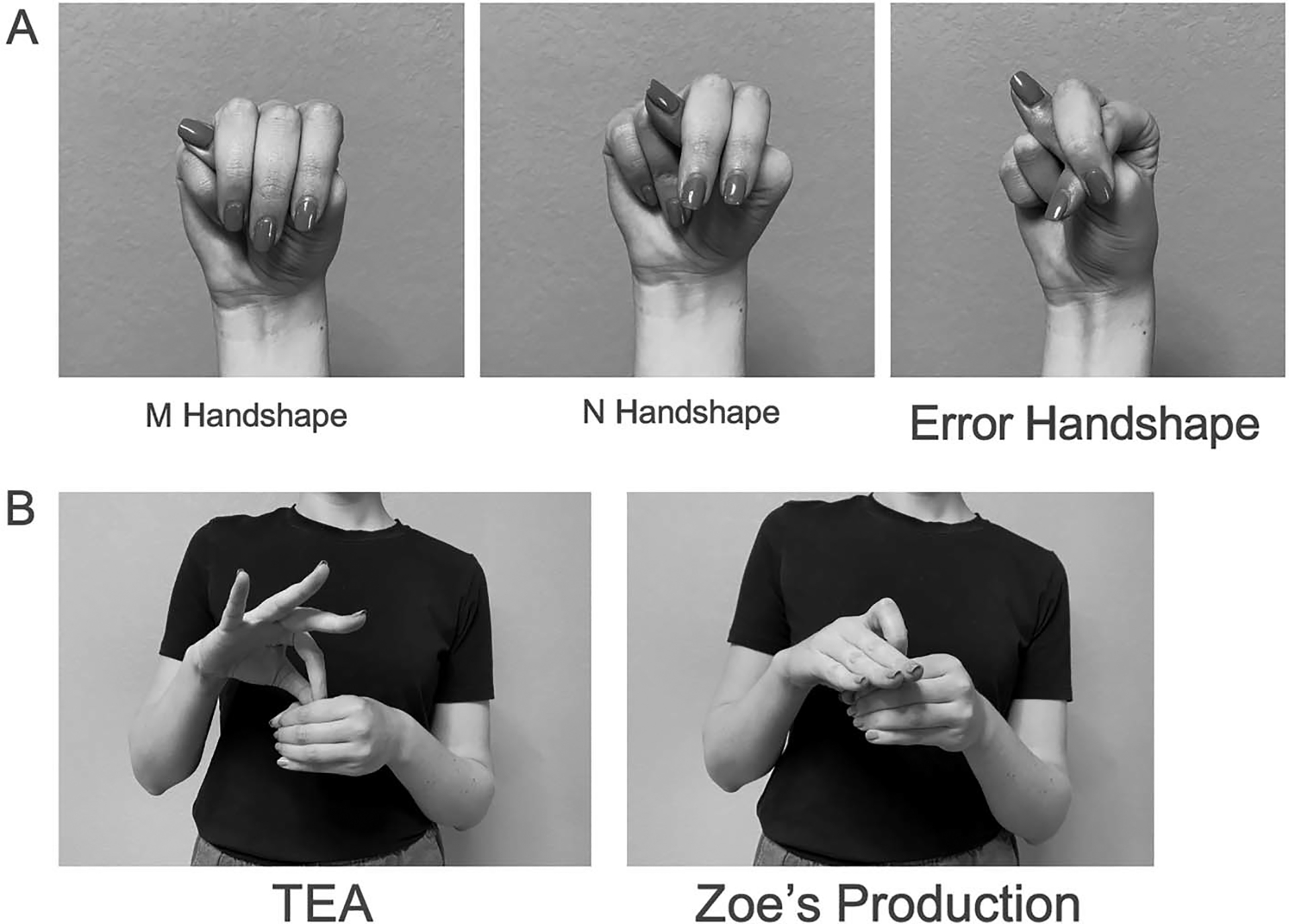
Examples of distortion errors when attempting to reproduce the letter signs M and N (A) and the sign TEA (B).

In CAS, a common symptom is distorted speech sounds (e.g., Shriberg et al., 2011), which are misarticulation errors, rather than sound substitutions. Similarly, Zoe produced handshape distortions. For example, when asked to repeat the signs for the letter “M” and the letter “N,” she produced handshape distortions that do not correspond to existing ASL handshapes (see Figure 5A; see also Figure 2). Thus, these were not substitution errors. Similarly, when attempting to reproduce the sign TEA, she produced the F handshape with the extended fingers contacting each other, rather than spread apart (see Figure 5B)—this is a distortion of the F handshape rather than substitution of another existing ASL handshape.

Children with CAS may also exhibit groping behaviors (e.g., Murray et al., 2015), where they appear to be searching for the correct placement or movement of their articulators before producing a sound or word. This can manifest as visible struggles, such as repeated attempts to move the articulators into the correct position before producing a sound. Zoe had a similar presentation in which she sometimes quickly changed her handshapes when trying to imitate a sign. For example, when trying to produce the two-handed sign DATE, she rapidly adjusted the handshapes on each hand (shifting between a 1 and a D handshape) before producing the correct sign (D handshapes on both hands). When imitating the sign NIECE, she initially produced it with a flat-O handshape and then changed it to a T handshape, which is closer to the target N handshape.

## Self-Manipulation of the Hands

Further evidence for deficits in motor planning comes from Zoe’s self-corrections in which she manipulates her own hands. For example, Zoe had difficulty imitating the sign THREE, and she switched to her left hand, using her right hand to move her fingers into the correct handshape (the thumb, index, and middle fingers are extended). When asked to imitate the sign for the letter I (only the pinky is extended), she produced a Y handshape (pinky and thumb extended) and then used her left hand to move her right thumb over to create the correct handshape. Throughout the imitation task, Zoe would often look at her own hand to make sure that her handshape matched the model’s handshape. Such self-awareness and self-corrections indicate that Zoe can correctly perceive the model’s productions and recognizes when her own articulation does or does not match her intended production. This pattern of performance indicates a deficit in motor planning, rather than a deficit in sign perception. This type of self-correction—overt manipulation of the linguistic articulators—is difficult for speakers (e.g., children cannot usefully manipulate their tongue with their hands) and, thus, this may be a distinctive marker for CASign.

In summary, Zoe’s sign articulation errors are consistent with an underlying deficit in motor planning and/or programming. Her articulation errors were unusual and unexpected, even when considering her language delay. Zoe’s error patterns suggested intact phonological representations of signs and an impairment in the ability to transform those representations into motor commands for production. Similar to CAS, Zoe produced inconsistent articulation errors, sign distortions, and groping behaviors. Possibly unique to CASign, Zoe unexpectedly produced location errors at the age of 4 years and manipulated her own hands to create well-formed signs.

## Discussion

To our knowledge, this is the first description of a possible case of CASign. Despite full access to ASL from her deaf parents and educational environment, Zoe makes frequent articulation errors in her signing, and these errors are inconsistent across signs. CAS is distinguished from a phonological disorder in that phonological impairments tend to involve consistent errors for a specific speech sound or set of speech sounds, although inconsistent phonological disorders can occur (Dodd et al., 2023). We suggest that CASign can be distinguished from a consistent sign-based phonological disorder. Zoe’s errors were not limited to particular phonological units (handshapes, movements, and locations) or their combination. Another parallel between CAS and CASign is that the child’s errors are not limited to motorically difficult articulations and can occur with early acquired phonological forms (i.e., unmarked segments or unmarked handshapes). Both types of apraxia present with articulation error patterns that are not observed in typical development, such as substituting a more complex form for a simpler form.

In addition, this case highlights some possible modality-specific characteristics of apraxia in the visual-manual modality. First, the production of location errors in a sign imitation task could be a possible marker of CASign for children older than 3 years, because typically developing children rarely, if ever, make such errors. Location errors are hypothesized to be rare (particularly for imitation tasks), because the gross motor skills to reach a target location on the body are in place early in development. The motor planning deficit associated with CASign could give rise to this type of error due to deficits in motor planning during language production (i.e., difficulty targeting a location on the body). Second, bimanual production errors (e.g., see Figure 4) are unique to sign language and involve the coordination of two large articulators. Third, the ability of the child to manipulate her own articulators and to directly observe her articulators is unique to the sign modality. Self-corrections that are made by manipulating the hands indicate that the child is aware of the target production but is not able to plan the motor movements appropriately (or may not be able to retrieve or combine the correct phonological representations).

Zoe’s case is distinct from two recently reported cases of native signing deaf children who exhibit sign language disorders. Kelley and McCann (2021) describe the case of “Lucas” (age 5 years) who, like Zoe, exhibited a language delay, but unlike Zoe did not have frank difficulties with articulation. Lucas had weaknesses in lexical diversity, use of third-person pronouns, and more complex syntax, but no significant phonological or articulation deficits were reported. Thus, DLDs in sign language can occur both with and without articulation disorders. Quinto-Pozos and Cooley (2020) reported the case of “Gregory” (studied between the ages of 11 and 13 years) who exhibited typical ASL comprehension ability and intact fine-motor skills, but had sign production difficulties. His sign productions were semantically and grammatically correct, but his intelligibility was poor due to consistent misarticulations. His misarticulations were not attributed to an underlying motor impairment or motor programming deficit, because his motor skills were considered intact based on school reports and informal assessments. Gregory had difficulty producing clear and fully formed signs, for example, failing to fully extend his fingers or to fully close his fist. These lax, partially formed articulations were not attributed to deficits in the underlying phonological representations of signs or to an inability to correctly retrieve or combine the phonological units of ASL, as would be expected for a sign-based phonological disorder. Rather, these errors might be indicative of sign dysarthria, which is characterized by lax, imprecise handshape productions (Tyrone, 2014). Quinto-Pozos and Cooley (2020) did not describe the same type of errors and behaviors that were observed for Zoe and that we suggest may be markers of CASign, for example, substituting a more complex handshape for a simpler handshape, producing fully articulated, but incorrect handshapes, and overtly manipulating the hands to form the correct handshape. Thus, the cases of Gregory and Zoe indicate that, as with speech, there may be more than one type of sign production disorder.

As noted in the first section, it is not uncommon for children with autism to also be diagnosed with CAS. However, Zoe demonstrates typical pragmatic language skills, which is not consistent with a diagnosis of autism. Nonetheless, Zoe made some types of sign-specific errors that have been observed for deaf signing children with autism (Shield et al., 2017; Shield & Meier, 2012). For example, Zoe made movement errors in which she reversed the direction of the sign’s movement. When imitating the sign CLASS, she produced an inward movement (toward herself), rather than an outward movement. Similarly, she reversed the movement direction for the sign SHEEP, producing an outward movement rather than an inward movement. These movement reversals are parallel to the movement reversals reported for deaf autistic signing children (Shield & Meier, 2012). In addition, Zoe’s contralateral location error (see Figure 1) is similar to location errors produced by autistic signing children, but such errors have been attributed to difficulties with perspective taking in the autistic children (Shield, 2010; Shield & Meier, 2018). That is, children with autism tend to reproduce signs as they see them from their perspective, without correctly reversing the location or movement. Typically developing children correctly reverse sign locations and movements by the end of their second year (Shield & Meier, 2018). It is possible that these sign-specific “reversal” errors can arise from either perspective-taking difficulties (with autism) or from a motor planning deficit (with CASign).

The possible existence of CASign raises questions regarding the use of manual signs and gestures as an augmentative and alternative communication (AAC) strategy for hearing children with severe apraxia of speech. Interestingly, some children with severe CAS are able to successfully produce manual signs and gestures to aid communication (Chenausky, Gagné, et al., 2022; Culp, 1989; Tierney et al., 2016). These findings suggest that CAS and CASign might be dissociable. Nonetheless, some children with CAS have difficulty learning signs, and sign language instruction is not always as successful as other types of AAC methods (e.g., Cumley & Swanson, 1999). More research is needed to determine the dissociability of CAS, limb apraxia, and CASign.

Finally, we know of no reports that clearly document a developmental phonological disorder in sign language, which makes it difficult to definitively distinguish between sign production deficits due to motor planning versus phonological impairment. However, based on psycholinguistic studies of sign production (for reviews, see Corina et al., 2014; Emmorey, 2023), we suggest that the clearest evidence of a phonological impairment would be phonemic-level errors, such as substitutions of similar handshapes, locations, and movements. Psycholinguistic evidence indicates that these phonological units are assembled during production and thus could be mis-selected or misrepresented. Furthermore, a phonological disorder might result in handshape errors that reflect the organization of handshape features, as reported for phonological paraphasias in signers with aphasia (Corina, 2000). For example, an incorrectly selected position feature (±Open) for the Selected Fingers of a handshape would result in a change in position of the remaining Unselected Fingers, due to a later redundancy rule that specifies the configuration of the Unselected Fingers (Corina, 2000). For example, one signer with aphasia produced an F handshape (−Open Selected Fingers) instead of an L handshape (+Open Selected Fingers). Corina (2000) argues that such errors are phonological and not phonetic distortions because they differ only in configuration features and can be traced to an error early in the phonological derivation. Zoe’s handshape errors cannot easily be explained as phonological feature errors of the type described by Corina (2000).

In summary, this case study suggests that CAS is not specific to the spoken modality, and this has important implications for clinicians supporting deaf and hard of hearing children. Professionals working in school settings with signing children do not have many formal assessments available to them, and our study of Zoe indicates that a clinician-created sign imitation task could be very useful to help identify CASign. Such an imitation task should include signs that have marked and unmarked handshapes, varied movements and location targets, and the task should include both phonologically simple and complex signs. Further work is needed to determine whether CAS and CASign tend to cooccur (as suspected in Zoe’s case) or whether these two types of apraxia can be dissociated. Finally, more studies of CASign and other developmental sign language disorders are needed to aid clinicians in recognizing and diagnosing these disorders and planning effective interventions.

## Data Availability

The data analyzed for the current study are not publicly available due to privacy concerns.

## Footnotes

1 Hyperlinks in this article link to videos of the ASL signs from the ASL-LEX database (Caselli et al., 2017; Sehyr et al., 2021).

## Appendix

Illustrations of ASL Handshapes

## References

[R1] Allen TE, & Enns C (2013). A psychometric study of the ASL Receptive Skills Test when administered to deaf 3-, 4-, and 5-year-old children. Sign Language Studies, 14(1), 58–79. 10.1353/sls.2013.0027

[R2] Allison KM, Cordella C, Iuzzini-Seigel J, & Green JR (2020). Differential diagnosis of apraxia of speech in children and adults: A scoping review. Journal of Speech, Language, and Hearing Research, 63(9), 2952–2994. 10.1044/2020_jslhr-20-00061 PMC7890226 32783767

[R3] American Speech-Language-Hearing Association. (2007). Childhood apraxia of speech [Technical report]. 10.1044/policy.ps2007-00277

[R4] Battison R (1978). Lexical borrowing in American Sign Language. Linstok Press.

[R5] Boyes Braem P (1990). Acquisition of the handshape in American Sign Language. In Volterra V & Erting CJ (Eds.), From gesture to language in hearing and deaf children (pp. 107–127). Springer-Verlag. 10.1007/978-3-642-74859-2_10

[R6] Brentari D (2019). Sign language phonology. Cambridge University Press. 10.1017/9781316286401

[R7] Caselli NK, Sehyr ZS, Cohen-Goldberg AM, & Emmorey K (2017). ASL-LEX: A lexical database of American Sign Language. Behavior Research Methods, 49(2), 784–801. 10.3758/s13428-016-0742-0 27193158 PMC5116008

[R8] Chenausky KV, Gagné D, Stipancic KL, Shield A, & Green JR (2022). The relationship between single-word speech severity and intelligibility in childhood apraxia of speech. Journal of Speech, Language, and Hearing Research, 65(3), 843–857. 10.1044/2021_JSLHR-21-00213 PMC9150686 35133873

[R9] Chenausky KV, Verdes A, & Shield A (2022). Concurrent predictors of supplementary sign use in school-aged children with childhood apraxia of speech. Language, Speech, and Hearing Services in Schools, 53(4), 1149–1160. 10.1044/2022_LSHSS-22-00017 36191130 PMC9913131

[R10] Conlin K, Mirus GR, Mauk C, & Meier RP (2000). Acquisition of first signs: Place, handshape, and movement. In Chamberlain C, Morford JP, & Mayberry RI (Eds.), Language acquisition by eye (pp. 51–70). Erlbalum. 10.4324/9781410601766-16

[R11] Corina DP (2000). Some observations regarding paraphasia in American Sign Language. In Emmorey K & Lane HL (Eds.), The signs of language revisited (pp. 414–426). Psychology Press. 10.4324/9781410604972-37

[R12] Corina DP, Gutierrez E, & Grosvald M (2014). Sign language production: An overview. In Goldrick M, Ferreira V, & Miozzo M (Eds.), The Oxford handbook of language production (pp. 393–416). Oxford University Press.

[R13] Culp D (1989). Developmental apraxia and augmentative or alternative communication—A case example. Augmentative and Alternative Communication, 5(1), 27–34. 10.1080/07434618912331274936

[R14] Cumley G, & Swanson S (1999). Augmentative and alternative communication options for children with developmental apraxia of speech: Three case studies. Augmentative and Alternative Communication, 15(2), 110–125. 10.1080/07434619912331278615

[R15] Dodd B, McIntosh B, Crosbie S, & Holm A (2023). Diagnosing inconsistent phonological disorder: Quantitative and qualitative measures. Clinical Linguistics & Phonetics, 1–24. 10.1080/02699206.2023.2224916 37382651

[R16] Emmorey K (2023). Sign production: Signing vs. speaking: How does the biology of linguistic expression affect production? In Hartsuiker R & Strijkers K (Eds.), Cognitive issues in the psychology of language (pp. 233–256). Routledge (Taylor & Francis).

[R17] Emmorey K, Borinstein HB, Thompson R, & Gollan TH (2008). Bimodal bilingualism. Bilingualism: Language and Cognition, 11(1), 43–61. 10.1017/s1366728907003203 19079743 PMC2600850

[R18] Glaspey A, & Stoel-Gammon C (2007). A dynamic approach to phonological assessment. Advances in Speech Language Pathology, 9(4), 286–296. 10.1080/14417040701435418

[R19] Gu S, Chen Pichler D, Kozak LV, & Lillo-Martin D (2022). Phonological development in American Sign Language-signing children: Insights from pseudosign repetition tasks. Frontiers in Psychology, 13. Article 921047. 10.3389/fpsyg.2022.921047 PMC9496651 36160535

[R20] Guiberson M, & Atkins J (2012). Speech-language pathologists’ preparation, practices, and perspectives on serving culturally and linguistically diverse children. Communication Disorders Quarterly, 33(3), 169–180. 10.1177/1525740110384132

[R21] Helm-Estabrooks N (1984). A discussion of apraxia, aphasia, and gestural language. American Journal of Physiology-Regulatory, Integrative and Comparative Physiology, 246(6), R884–R887. 10.1152/ajpregu.1984.246.6.R884 6742162

[R22] Hrastelj L, & Knight R-A (2017). Ingressive speech errors: A service evaluation of speech-sound therapy in a child aged 4;6. International Journal of Language & Communication Disorders, 52(4), 479–488. 10.1111/1460-6984.12287 27891743

[R23] Iuzzini-Seigel J, Moorer L, & Tamplain P (2022). An investigation of developmental coordination disorder characteristics in children with childhood apraxia of speech. Language, Speech, and Hearing Services in Schools, 53(4), 1006–1021. 10.1044/2022_LSHSS-21-00163 36041512

[R24] Kelley LE, & McCann JP (2021). Language intervention isn’t just spoken: Assessment and treatment of a deaf signing child with specific language impairment. Language, Speech, and Hearing Services in Schools, 52(4), 978–992. 10.1044/2021_lshss-21-00038 34618545

[R25] Lewis BA, Freebairn LA, Hansen AJ, Iyengar SK, & Taylor HG (2004). School-age follow-up of children with childhood apraxia of speech. Language, Speech, and Hearing Services in Schools, 35(2), 122–140. 10.1044/0161-1461(2004/014) 15191325

[R26] Lillo-Martin D, de Quadros RM, Chen Pichler D, & Fieldsteel Z (2014). Language choice in bimodal bilingual development. Frontiers in Psychology, 5, Article 1163. 10.3389/fpsyg.2014.01163 PMC4202712 25368591

[R27] Mann W, Hoskin J, & Dumbrill H (2022). Dynamic assessment of learners of a sign language. In Haug T, Mann W, & Knoch U (Eds.), The handbook of language assessment across modalities. Oxford University Press. 10.1093/oso/9780190885052.003.0009

[R28] Mann W, Marshall CR, Mason K, & Morgan G (2010). The acquisition of sign language: The impact of phonetic complexity on phonology. Language Learning and Development, 6(1), 60–86. 10.1080/15475440903245951

[R29] Marentette P, & Mayberry RI (2000). Principles for an emerging phonological system: A case study of early ASL acquisition. In Chamberlain C, Morford JP, & Mayberry RI (Eds.), Language acquisition by eye (pp. 71–90). Erlbaum. 10.4324/9781410601766

[R30] Mason K, Rowley K, Marshall CR, Atkinson JR, Herman R, Woll B, & Morgan G (2010). Identifying specific language impairment in deaf children acquiring British Sign Language: Implications for theory and practice. British Journal of Developmental Psychology, 28(1), 33–49. 10.1348/026151009x484190 20306624

[R31] Meier RP, Mauk CE, Cheek A, & Moreland CJ (2008). The form of children’s early signs: Iconic or motoric determinants? Language learning and development, 4(1), 63–98. 10.1080/15475440701377618

[R32] Morgan G, Barrett-Jones S, & Stoneham H (2007). The first signs of language: Phonological development in British Sign Language. Applied Psycholinguistics, 28(1), 3–22. 10.1017/S0142716407070014

[R33] Morgan H, Novogrodsky R, & Sandler W (2019, September 26-28). Phonological complexity and frequency in the lexicon: A quantitative crosslinguistic study [Paper presentation]. TISLR13: Theoretical Issues in Sign Language Research, Hamburg, Germany.

[R34] Murray E, McCabe P, Heard R, & Ballard KJ (2015). Differential diagnosis of children with suspected childhood apraxia of speech. Journal of Speech, Language, and Hearing Research, 58(1), 43–60. 10.1044/2014_JSLHR-S-12-0358 25480674

[R35] Petitto LA, & Marentette PF (1991). Babbling in the manual mode: Evidence for the ontogeny of language. Science, 251(5000), 1493–1496. 10.1126/science.2006424 2006424

[R36] Poizner H, Bellugi U, & Iragui V (1984). Apraxia and aphasia for a visual-gestural language. American Journal of Physiology-Regulatory, Integrative and Comparative Physiology, 246(6), R868–R883. 10.1152/ajpregu.1984.246.6.R868 6742161

[R37] Quinto-Pozos D, & Cooley F (2020). A developmental disorder of signed language production in a native deaf signer of ASL. Language, 5(4), Article 40. 10.3390/languages5040040

[R38] Quinto-Pozos D, Singleton JL, & Hauser PC (2017). A case of specific language impairment in a deaf signer of American Sign Language. The Journal of Deaf Studies and Deaf Education, 22(2), 204–218. 10.1093/deafed/enw074 27884866

[R39] Rosenbek JC, & Wertz T (1972). A review of fifty cases of developmental apraxia of speech. Language, Speech, and Hearing Services in Schools, 3(1), 23–33. 10.1044/0161-1461.0301.23

[R40] Sehyr ZS, Caselli N, Cohen-Goldberg AM, & Emmorey K (2021). The ASL-LEX 2.0 Project: A database of lexical and phonological properties for 2,723 signs in American Sign Language. The Journal of Deaf Studies and Deaf Education, 26(2), 263–277. 10.1093/deafed/enaa038 33598676 PMC7977685

[R41] Shield A (2010). The signing of deaf children with autism: Lexical phonology and perspective-taking in the visual-spatial modality. [Unpublished doctoral dissertation]. University of Texas at Austin.

[R42] Shield A, Knapke K, Henry M, Srinivasan S, & Bhat A (2017). Impaired praxis in gesture imitation by deaf children with autism spectrum disorder. Autism & Developmental Language Impairments, 2. 10.1177/2396941517745674

[R43] Shield A, & Meier RP (2012). Palm reversal errors in native-signing children with autism. Journal of Communication Disorders, 45(6), 439–454. 10.1016/j.jcomdis.2012.08.004 22981637 PMC3479340

[R44] Shield A, & Meier RP (2018) Learning an embodied visual language: Four imitation strategies available to sign learners. Frontiers in Psychology, 9, Article 811. 10.3389/fpsyg.2018.00811 PMC5988899 29899716

[R45] Shriberg LD, Potter NL, & Strand EA (2011). Prevalence and phenotype of childhood apraxia of speech in youth with galactosemia. Journal of Speech, Language, and Hearing Research, 54(2), 487–519. 10.1044/1092-4388(2010/10-0068) PMC3070858 20966389

[R46] Siedlecki T, & Bonvillian JD (1993). Location, handshape & movement: Young children’s acquisition of the formational aspects of American Sign Language. Sign language studies, 78(1), 31–52. 10.1353/sls.1993.0016

[R47] Strand EA, & McCauley RJ (2019). Dynamic evaluation of motor speech skill (DEMSS) manual. Brookes Publishing.

[R48] Terband H, Maassen B, Guenther FH, & Brumberg J (2009). Computational neural modeling of speech motor control in childhood apraxia of speech (CAS). Journal of Speech, Language, and Hearing Research, 52(6), 1595–1609. 10.1044/1092-4388(2009/07-0283) PMC2959199 19951927

[R49] Tierney CD, Pitterle K, Kurtz M, Nakhla M, & Todorow C (2016). Bridging the gap between speech and language: Using multimodal treatment in a child with apraxia. Pediatrics, 138(3), Article e20160007. 10.1542/peds.2016-0007 27492818

[R50] Tyrone ME (2014). Sign dysarthria: A speech disorder in signed language. In Quinto-Pozos D (Ed.), Multilingual aspects of signed language communication and disorder (pp. 162–185). Multilingual Matters. 10.21832/9781783091317-010

